# Tanshinone IIA alleviates brain damage in a mouse model of neuromyelitis optica spectrum disorder by inducing neutrophil apoptosis

**DOI:** 10.1186/s12974-020-01874-6

**Published:** 2020-06-25

**Authors:** Ye Gong, Ya-ling Zhang, Zhen Wang, Huan-huan Song, Yuan-chu Liu, Ao-wei Lv, Li-li Tian, Wen-li Zhu, Ying Fu, Xiao-li Ding, Lang-jun Cui, Ya-ping Yan

**Affiliations:** 1 Key Laboratory of the Ministry of Education for Medicinal Resources and Natural Pharmaceutical Chemistry, National Engineering Laboratory for Resource Development of Endangered Crude Drugs in Northwest of China, College of Life Sciences, Shaanxi Normal University, No. 620, West Chang’an Avenue, Xi’an, 710119 China; 2 Department of Neurology, Xuanwu Hospital, Capital Medical University, No.45, Changchun Street, Beijing, 100053 China; 3 Department of Neurology, Tianjin Neurological Institute, Tianjin Medical University General Hospital, No.154, Anshan Road, Tianjin, 300052 China

**Keywords:** Tanshinone IIA, Neuromyelitis optica spectrum disorder, Mouse model, Neuroinflammation, Neutrophil apoptosis

## Abstract

**Background:**

Neuromyelitis optica spectrum disorder (NMOSD), an autoimmune astrocytopathic disease associated with the anti-aquaporin-4 (AQP4) antibody, is characterized by extensive necrotic lesions primarily located on the optic nerves and spinal cord. Tanshinone IIA (TSA), an active natural compound extracted from *Salvia miltiorrhiza* Bunge, has profound immunosuppressive effects on neutrophils.

**Objective:**

The present study aimed to evaluate the effect of TSA on NMOSD mice and explore the underlying mechanisms. Mice were initially administered TSA (pre-TSA group, *n* = 20) or vehicle (vehicle group, *n* = 20) every 8 h for 3 days, and then NMOSD model was induced by intracerebral injection of NMOSD-immunoglobulin G (NMO-IgG) and human complement (hC). In addition, post-TSA mice (*n* = 10) were administered equal dose of TSA at 8 h and 16 h after model induction. At 24 h after intracerebral injection, histological analysis was performed to assess the inhibitory effects of TSA on astrocyte damage, demyelination, and neuroinflammation in NMOSD mice, and western blotting was conducted to clarify the effect of TSA on the NF-κB and MAPK signaling pathways. Furthermore, flow cytometry and western blotting were conducted to verify the proapoptotic effects of TSA on neutrophils in vitro.

**Results:**

There was a profound reduction in astrocyte damage and demyelination in the pre-TSA group and post-TSA group. However, prophylactic administration of TSA induced a better effect than therapeutic treatment. The number of infiltrated neutrophils was also decreased in the lesions of NMOSD mice that were pretreated with TSA. We confirmed that prophylactic administration of TSA significantly promoted neutrophil apoptosis in NMOSD lesions in vivo, and this proapoptotic effect was mediated by modulating the caspase pathway in the presence of inflammatory stimuli in vitro. In addition, TSA restricted activation of the NF-κB signaling pathway in vivo.

**Conclusion:**

Our data provide evidence that TSA can act as a prophylactic agent that reduces NMO-IgG-induced damage in the mouse brain by enhancing the resolution of inflammation by inducing neutrophil apoptosis, and TSA may serve as a promising therapeutic agent for neutrophil-associated inflammatory disorders, such as NMOSD.

## Introduction

Neuromyelitis optica spectrum disorder (NMOSD), formerly referred to as NMO or Devic’s disease, is an astrocyte-specific central nervous system (CNS) disorder characterized by relapsing optic neuritis and longitudinally extensive transverse myelitis which is a secondary phenomenon [1–5]. Astrocyte damage is much more striking than myelin and neuron damage in NMOSD compared with multiple sclerosis, and autoimmune astrocytopathy is the primary pathology of NMOSD [5]. Approximately 70–90% of NMOSD patients are seropositive for disease-specific autoantibodies targeting the water channel protein aquaporin-4 (AQP4) [6–8], and AQP4 is mainly localized to astrocytic end-feet that are adjacent to microvessels at the blood-brain barrier (BBB) throughout the CNS [9, 10]. The presence of AQP4-immunoglobulin G (AQP4-IgG) is a serological or laboratory hallmark of NMOSD that is used to distinguish NMOSD from relapsing-remitting multiple sclerosis (RRMS) [11].

There is growing evidence that NMO-IgG/AQP4-IgG is pathogenic in NMOSD [3, 12–15] by a mechanism involving complement-dependent cytotoxicity (CDC) [16, 17] and antibody-dependent cell-mediated cytotoxicity (ADCC) [18, 19], which lead to astrocyte damage and an inflammatory response, causing oligodendrocyte injury, demyelination, and neurological deficits. Neutrophils play an important role in the formation of NMOSD lesions [20, 21]. The severity of NMOSD lesions is increased in mice with neutrophilia and reduced in mice with neutropenia [21]. The pathogenetic implication of neutrophils in NMOSD was demonstrated in a previous report of a patient whose first NMOSD episode was worsened by inadvertent administration of granulocyte-colony stimulating factor (G-CSF) [22].

Experimental NMOSD models are needed to elucidate the underlying pathophysiological mechanisms of this disease and to test candidate therapeutic drugs. Other groups and our group have fully confirmed that intracerebral injection of NMO-IgG and human complement (hC) in mice are sufficient to induce NMOSD-like lesions [23–26].

**Fig. 1:**
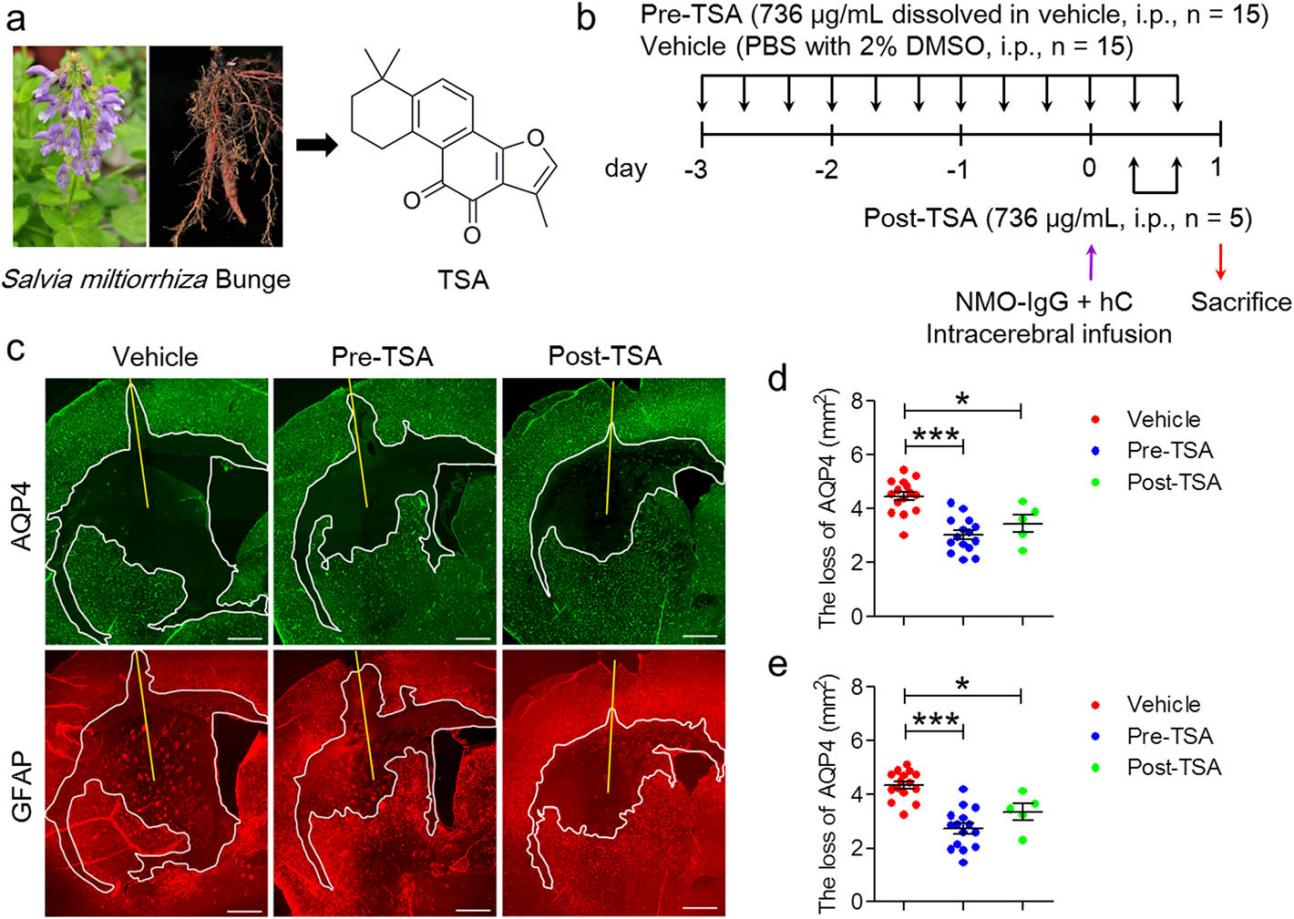
TSA administration notably suppresses astrocyte damage in NMOSD lesions at 24 h after brain injection. **a***Salvia miltiorrhiza* Bunge and the chemical structures of TSA. **b** The protocol of the animal experiment. The purple arrow indicates the time point of NMO-IgG and hC injection. The black arrows represent the time points of TSA or vehicle treatment. The red arrow indicates the end point of the animal model experiment. **c** Representative immunofluorescence staining of AQP4 (*top-left*, green) and GFAP (*bottom-left*, red) in TSA- or vehicle-treated mice. **d** Reduction in the area of astrocyte marker immunoreactivity. Vehicle, *n* = 15; pre-TSA, *n* = 15; post-TSA, *n* = 5. *p < 0.05, ***p < 0.001. The data are representative of three independent experiments. One-way ANOVA followed by Tukey’s multiple comparison tests was performed

Tanshinone IIA (TSA, Fig. 1a), an active natural compound extracted from *Salvia miltiorrhiza* Bunge (Fig. 1a) [27], has been clinically used to treat cardiovascular [28, 29] and cerebrovascular [30–32] diseases. These protective effects were attributed at least in part to its anti-inflammatory properties [28–31]. TSA can accelerate the resolution of inflammation by promoting neutrophil transmigration and apoptosis in zebrafish [33]. TSA also efficiently ameliorates rheumatoid arthritis in mice by inhibiting neutrophil infiltration and activation and by promoting neutrophil apoptosis in the ankle joints [34].

Here, we utilized an NMOSD mouse model to evaluate the effect of TSA on NMOSD and the underlying active mechanism. Our results indicated that prophylactic administration of TSA significantly suppressed astrocyte damage and demyelination in NMOSD mice, and its protective effect was correlated with the degree of neutrophil apoptosis within lesions. We further confirmed that TSA indeed accelerates neutrophil apoptosis under inflammatory stimuli in vitro, and this proapoptotic effect was also observed in human neutrophils. These results suggest that TSA might serve as a prophylactic treatment for human neutrophil-associated inflammatory disorders.

## Methods

### Reagents

TSA (purity: ≥ 97%, catalog T4952), lipopolysaccharide (LPS, purity ≥ 97%, catalog L6143), and DMSO were purchased from Sigma-Aldrich. DyLight 488-conjugate donkey anti-rat IgG (catalog 712-545-153), DyLight 594-conjugate donkey anti-rat IgG (catalog 712-585-153), DyLight 488-conjugate donkey anti-rabbit IgG (catalog 711-545-152), and DyLight 594-conjugate donkey anti-goat IgG (catalog 705-585-147) were obtained from Jackson ImmunoResearch.

### Animals

Adult female C57BL/6 mice, 8–10 weeks of age, were purchased from Beijing HFK Bioscience Co., Ltd. The mice were maintained in standard housing cages under specific pathogen-free conditions. All procedures were approved by the Committee for Research and Animal Ethics of Shaanxi Normal University and were conducted in accordance with the US Public Health Service’s Policy on the Humane Care and Use of Laboratory Animals. Investigators involved in pathological staining and analysis were blinded to the experimental groups during the experiments.

### Isolation of NMO-IgG

The total IgG from NMOSD patient sera was purified as previously described [24, 25]. Serum was obtained from five patients (P1–P5) with an established diagnosis of NMOSD and strong AQP4 autoantibody titers (AQP4-IgG titers ≤ 1:100). The clinical details of the patients with NMOSD were previously described [24]. Patient serum IgG was purified with protein-A resin (GeneScript, catalog: L00210) and eluted with 0.1 M glycine buffer (pH 2.8) and then neutralized in Tris buffer (1.0 M, pH 9.0). Finally, the samples were concentrated using Amicon ultra centrifugal filter units (100 kDa, Merck Millipore, catalog: UFC910008) to obtain NMO-IgG (15 mg/mL). Informed consent was obtained from all participants, and the study was approved by the Shaanxi Normal University Institutional Review Boards and Ethics Committee and was performed in accordance with the 1964 Helsinki declaration and its later amendments or comparable ethical standards.

### Induction of mouse NMOSD models and administration of TSA

First, mice in the prophylactic group (pre-TSA, *n* = 20) were administered TSA (736 μg/kg/day, dissolved in PBS with 2% DMSO), and mice in the control group mice (*n* = 20) were administered vehicle (PBS with 2% DMSO) by intraperitoneal injection (i.p.) every 8 h for 3 days (Fig. 1b). Then, the NMOSD model was induced based on previous studies [24, 25], and TSA or vehicle was administered by i.p. until 16 h after NMOSD model induction. In addition, therapeutic group (post-TSA) mice were administered an equal dose of TSA at 8 h and 16 h after injection of NMO-IgG plus hC (*n* = 10). Briefly, all animals were anesthetized with 10% chloral hydrate (3 mL/kg, i.p.) and mounted on a stereotactic frame (RWD Life Science). Following a midline scalp incision, a burr hole with a diameter of 1 mm was made in the skull 2 mm to the right of the bregma. A 33-gauge needle attached to a 25-μL gas-tight glass syringe (Hamilton) was inserted 3 mm deep to infuse 6 μL of NMO-IgG and 4 μL of hC (Innovative Research, catalog: IPLA-CSER) in a total volume of 10 μL (at 1 μL/min). After 24 h, the brains were processed into frozen sections.

### Immunofluorescence staining

Brain sections (8 μM) were permeabilized with ice-cold acetone for 7 min. After being blocked with 5% BSA (Sigma-Aldrich) in PBS, the sections were incubated with primary antibodies against AQP4 (1:100, rabbit polyclonal antibody, Santa Cruz Biotechnology, catalog: sc-20812), glial fibrillary acidic protein (GFAP, 1:1000, goat polyclonal antibody, Abcam, catalog: ab53554), myelin basic protein (MBP, 1:200, rat monoclonal antibody, clone: 12, Merck Millipore, catalog: MAB1965), CD45 (1:100, rat monoclonal antibody, clone 30-F11, BD Pharmingen, catalog 550539), Ly6G (1:100, rat monoclonal antibody, clone 1A8, BD Pharmingen, catalog 551459), and ionized calcium binding adapter molecule 1 (Iba-1, 1:500, rabbit polyclonal antibody, Wako, catalog 019-19741) at 4 °C overnight. The sections were washed three times with ice-cold PBS and incubated with the corresponding secondary antibody at room temperature for 60 min. Finally, the slides were covered with Fluoroshield mounting medium with DAPI (Abcam, catalog: ab104139). The results were visualized using a fluorescence microscope (Leica DM6000B). Coronal brain sections through the needle tract were selected, and areas were defined by hand and quantified using the ImageJ software (NIH).

### Analysis of neutrophil apoptosis in vivo

To analyze the extent of neutrophil apoptosis in the lesions, brain tissues were assessed by terminal deoxynucleotidyl transferase (TdT)-mediated biotin-dUTP nick end labeling (TUNEL) staining using an in situ cell death detection kit (Roche Diagnostics, catalog 11-684-795-910) combined with Ly6G immunofluorescence staining according to the manufacturer’s instructions. The results were visualized by a Leica DM6000B fluorescence microscope.

### Murine BMNE isolation and culture

Murine bone marrow-derived neutrophil (BMNE) isolation was performed as previously described [35]. Briefly, femurs and tibias were removed and flushed with Hank’s balanced salt solution (HBSS) without Ca^2+^/Mg^2+^. The BM cells were suspended in HBSS and overlaid on top of a 2-layer Percoll (GE Healthcare, catalog 17089101) gradient (72% and 65% in HBSS) and then centrifuged at 1200×*g* for 30 min at 25 °C. BMNEs were recovered at the interface of the 65–72% fractions, stained using antibody against APC-CD45 (clone 30-F11, eBioscience, catalog 17-0451-82), PE-Ly6G (clone 1A8-Ly6G, eBioscience, catalog 12-9668-80), Alexa Flour 488-CD11b (clone: M1/70, BioLegend, catalog 101219), and confirmed by flow cytometry (purity = 90.93%, Fig. S1a). The neutrophils were washed twice, and then cultured in RPMI 1640 complete medium at 37 °C with 5% CO_2_.

### Human primary neutrophil and PBMC isolation

Primary human neutrophils were obtained from anticoagulated venous blood (treated with EDTA) from healthy volunteers (male, *n* = 2; female, *n* = 3; 24–29 years old) who had not taken any drugs for at least 2 weeks before the experiments. Informed consent was obtained from all participants, and the study was approved by the Shaanxi Normal University Institutional Review Boards and Ethics Committee and was performed in accordance with the 1964 Helsinki declaration and its later amendments or comparable ethical standards. The blood was used within 2 h of drawing from the donor. Human neutrophils were isolated by density gradient centrifugation (500×*g* for 30 min) using Polymorphprep (Axis-Shield) [36]. After centrifugation, the upper band of peripheral blood mononuclear cells (PBMCs) and the lower band of polymorphonuclear cells (PMNs) were harvested. Residual of erythrocytes were eliminated using red blood cell lysing buffer (BD Bioscience, catalog 555899) at room temperature for 5 min. Then, the process was ceased by adding RPMI 1640 basic medium, and the mixture was centrifuged at 250×*g* for 10 min at 4 °C. The purity of the isolated neutrophils was > 99%, as determined by flow cytometry using FITC-conjugated anti-human CD15 antibody (clone: HI98, BioLegend, catalog 301903) staining (Fig. S1b). Freshly isolated cells were then cultured in RPMI 1640 complete medium at 37 °C with 5% CO_2_.

### Neutrophil apoptosis analysis by flow cytometry

Human or murine neutrophils (1 × 10^6^ cells/mL/well) were plated onto 24-well plates and cultured with 100 ng/mL LPS in the absence or presence of TSA (1 μM, 5 μM, and 10 μM) at 37 °C with 5% CO_2_. The cells were harvested at the indicated time points and washed twice with cell staining buffer. Then, the cells were suspended in 500 μL of binding buffer and incubated with 5 μL of APC-Annexin V and 10 μL of propidium iodide (PI) (BioLegend, catalog 640932) at room temperature for 15 min in the dark. Flow cytometry analysis was performed using a NovoCyte flow cytometer (ACEA). At least 3 × 10^4^ events were recorded and analyzed using the FlowJo software (Tree Star). In all analyses, cell debris was eliminated by appropriate forward and side scatter gating.

### Western blotting

The mice were anesthetized and perfused with 40 mL of PBS. Then, the hemispheres containing the lesions were homogenized in NP40 cell lysis buffer (Thermo Fisher, catalog: FNN0021) containing EDTA-free complete protease inhibitor cocktail tablets (Roche, catalog 11873580001) and a phosphatase inhibitor cocktail (04906845001) at 4 °C.

For in vitro culture cells, neutrophils were harvested at the indicated time points. The cells were washed twice with ice-cold PBS and lysed with NP40 cell lysis buffer containing EDTA-free complete protease inhibitor cocktail tablets and a phosphatase inhibitor cocktail at 4 °C.

Cell lysates were centrifuged at 16,000*g* for 20 min at 4 °C. After centrifugation, the cell lysis supernatant was collected, mixed with SDS loading buffer, and boiled for 10 min. Total protein (30 μg) was resolved on 12% sodium dodecyl sulfate-polyacrylamide gels (SDS-PAGEs) and transferred to 0.45 μM polyvinylidene fluoride (PVDF) membranes (Merck Millipore) using a wet transfer method. Then, the membranes were washed twice with Tris-buffered saline with 0.05% Tween-20 (TBST) and blocked with 5% skim milk (Difco) for 1 h at room temperature followed by incubation with rabbit anti-phosphorylated p65 (p-p65) polyclonal antibody (1:1000, Cell Signaling Technology, catalog 3033 T), rabbit anti-total p65 (t-p65) monoclonal antibody (1:1000, Cell Signaling Technology, catalog 8242 T), rabbit anti-phosphorylated p38 mitogen-activated protein kinase (p-p38 MAPK) monoclonal antibody (1:1000, Cell Signaling Technology, catalog 9211S), rabbit anti-total p38 MAPK (t-p38 MAPK) monoclonal antibody (1:1000, Cell Signaling Technology, catalog 9212S), rabbit anti-phosphorylated extracellular-regulated protein kinases 1/2 (p-Erk1/2) monoclonal antibody (1:1000, Cell Signaling Technology, catalog 4377), rabbit anti-total Erk1/2 (t-Erk1/2) polyclonal antibody (1:1000, Cell Signaling Technology, catalog 9102), rabbit anti-caspase-3 polyclonal antibody (1:1000, Proteintech, catalog 19677-1-AP), and rabbit anti-β-actin polyclonal antibody (1:1000, Bioss, catalog: bs-0061R) overnight at 4 °C. The membranes were washed three times with TBST and incubated with horseradish peroxidase-conjugated goat anti-rabbit (1:3000, Bioss, catalog: bs-0295G-HRP) secondary antibody. Then, the target proteins were visualized with ECL blotting reagents (GE Healthcare, catalog: RPN2109) by a digital gel image analysis system (Tanon 4600).

### Statistical analysis

Statistical analysis was performed using the GraphPad Prism 5.0 software. The values are presented as the mean ± standard error of the mean (SEM). When two groups were compared, the nonparametric Mann-Whitney test was used. One-way analysis of variance (ANOVA) followed by Tukey’s posttest was performed to analyze results from more than two groups. A *p* value < 0.05 was deemed statistically significant.

## Results

### TSA administration dramatically decreases astrocyte damage in NMOSD mice

To assess astrocyte damage 24 h after brain injection, AQP4 and GFAP immunofluorescence staining was performed. We found extensive loss of AQP4 and GFAP immunoreactivity in the basal ganglia and corpus callosum around the injection site 24 h after intracerebral injection of NMO-IgG and hC (Fig. 1c). The reduction in the area of AQP4 immunoreactivity was comparable to the reduction in the area of GFAP immunoreactivity in each group (4.47 ± 0.21 mm^2^ versus 4.34 ± 0.23 mm^2^ in vehicle-treated mice, 2.96 ± 0.26 mm^2^ versus 2.67 ± 0.24 mm^2^ in pre-TSA-treated mice, 3.45 ± 0.32 versus 3.35 ± 0.30 in post-TSA-treated mice) (Fig. 1d). Reactive gliosis was generally observed around the lesion (Fig. 1c *bottom*). Compared with the vehicle group, the two groups of TSA-treated NMOSD mice had dramatically reduced loss of AQP4 and GFAP, and the pre-TSA group displayed more obvious reduction in AQP4 and GFAP loss than the post-TSA group (Fig. 1d *right* and Fig. 1e *right*). These results suggest that TSA has a potent protective effect on astrocytes after NMO-IgG-induced hC activation in the brain, and prophylactic administration seems to be a better strategy than posttreatment.

### TSA suppresses demyelination in NMOSD mice

**Fig. 2:**
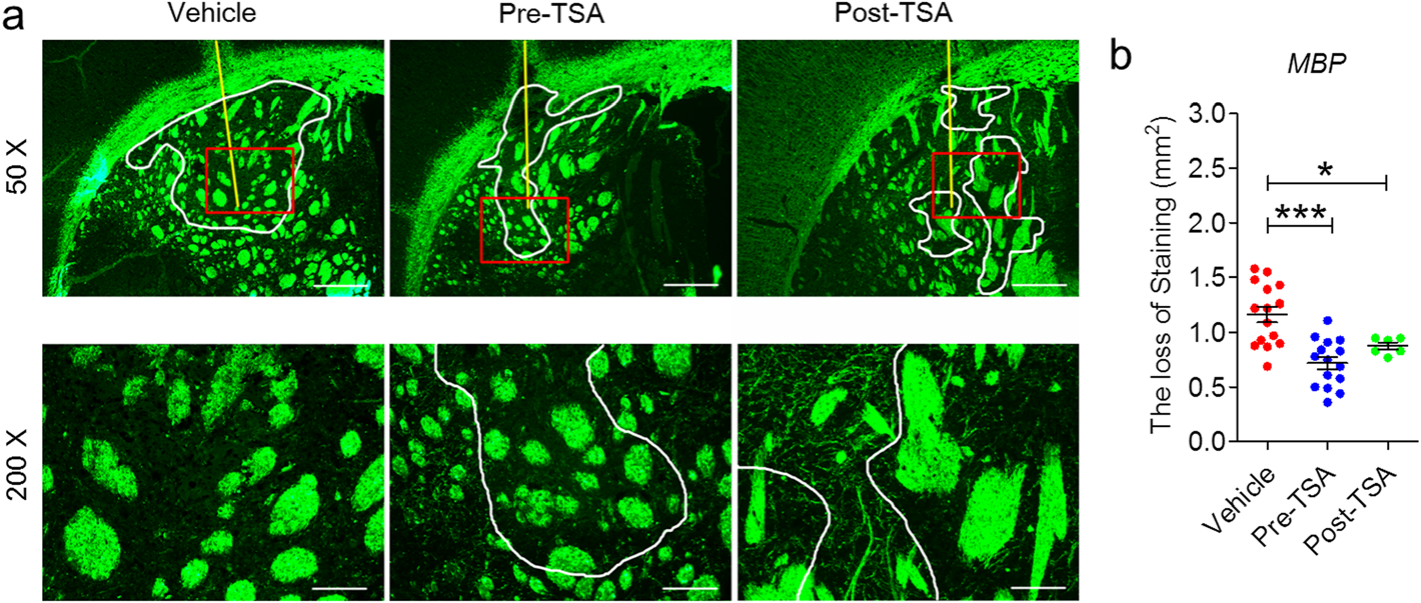
TSA administration inhibits the loss of myelination in NMOSD lesions at 24 h after brain injection. **a** Representative immunofluorescence staining of MBP. Lower magnification: *top*. Higher magnification: *bottom*. Yellow line, needle tract; white lines delimit the lesion. **b** Quantification of the extent of demyelination is expressed as the absolute reduction in area (mm^**2**^) of MBP in NMOSD lesions. Vehicle, *n* = 15; pre-TSA, *n* = 15; post-TSA, *n* = 5. *p < 0.05, ***p < 0.001. The data are representative of three independent experiments. One-way ANOVA followed by Tukey’s multiple comparison tests was performed

To analyze the alterations in myelin integrity in NMOSD mice upon TSA treatment, we performed MBP immunofluorescence staining of brain tissue. The injection site in all mice displayed disorganized myelinating fibers (Fig. 2a). The area of demyelination in TSA-treated NMOSD mice was notably reduced compared with that in mice treated with vehicle (from 1.16 ± 0.07 to 0.72 ± 0.10 mm^2^ for pre-TSA or 0.88 ± 0.03 mm^2^ for post-TSA Fig. 2b). Interestingly, the suppressive effect of TSA on demyelination appeared to be more effective in the prevention group than in the posttreatment group, but there was no significant difference between the pre-TSA and post-TSA groups (Fig. 2). This finding indicates that TSA may play an important role in maintaining myelin integrity after intracerebral injection of NMO-IgG and hC.

### TSA restricts neuroinflammation in the brains of NMOSD mice

**Fig. 3:**
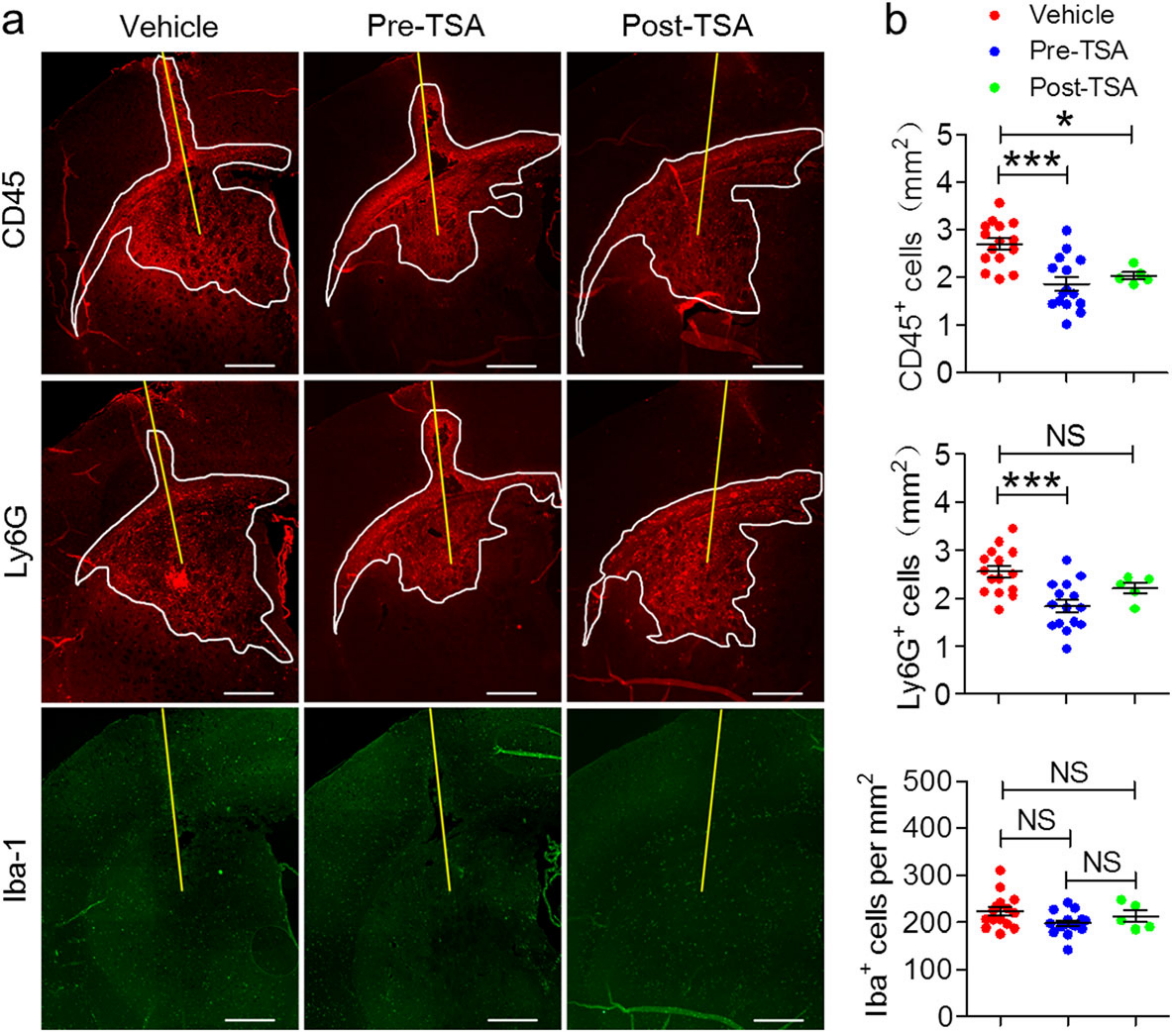
TSA administration markedly ameliorates neuroinflammation in NMOSD lesions at 24 h after brain injection. **a** Representative immunofluorescence staining of CD45^+^ leukocytes (*top*, red), Ly6G^+^ neutrophils (*middle*, red), and Iba^+^-activated microglia and macrophages (*bottom*, green) in the NMOSD lesion-side hemisphere at 24 h after brain injection. Yellow lines indicate needle tract; white lines delimit the lesion. **b** Inflammatory cells’ quantification is expressed as the absolute area (mm^**2**^) [CD45^+^ leukocytes (*top*) and Ly6G^+^ neutrophils (*middle*)] or the number of inflammatory cell subsets [Iba-1^+^-activated microglia and macrophages (*bottom*)] in the NMOSD lesion-side hemisphere. Vehicle, *n* = 15; pre-TSA, *n* = 15; post-TSA, n = 5. *p < 0.05, ***p < 0.001. The data are representative of three independent experiments. One-way ANOVA followed by Tukey’s multiple comparison tests was performed

We showed that intracerebral injection of NMO-IgG and hC caused extensive neuroinflammation. High numbers of CD45^+^ cells were observed around the injection site at 24 h after intracerebral injection of NMO-IgG and hC (Fig. 3a *top*). In addition, we found that neutrophils were the dominant cell type among these inflammatory cells (Ly6G^+^, red; Fig. 3a *middle*). Compared with mice treated with vehicle, TSA-treated mice exhibited a profound reduction in the number of CD45^+^ cells (Fig. 3b *top*). Pre-TSA-treated mice also exhibited reduced infiltration of Ly6G^+^ neutrophils (Fig. 3b *middle*). Furthermore, mild microglia/macrophages (Iba^+^, green; Fig. 3a *bottom*) activation was observed around the NMOSD lesions, but there were no significant differences in the numbers of microglia/macrophages in the three groups (Fig. 3b *bottom*). These results suggest that TSA is capable of inhibiting inflammation in the acute stage of NMOSD mice for at least 24 h, and prophylactic TSA administration seems suppress neutrophils inflammation.

### TSA accelerates recruited neutrophils apoptosis in the brains of NMOSD mice

**Fig. 4:**
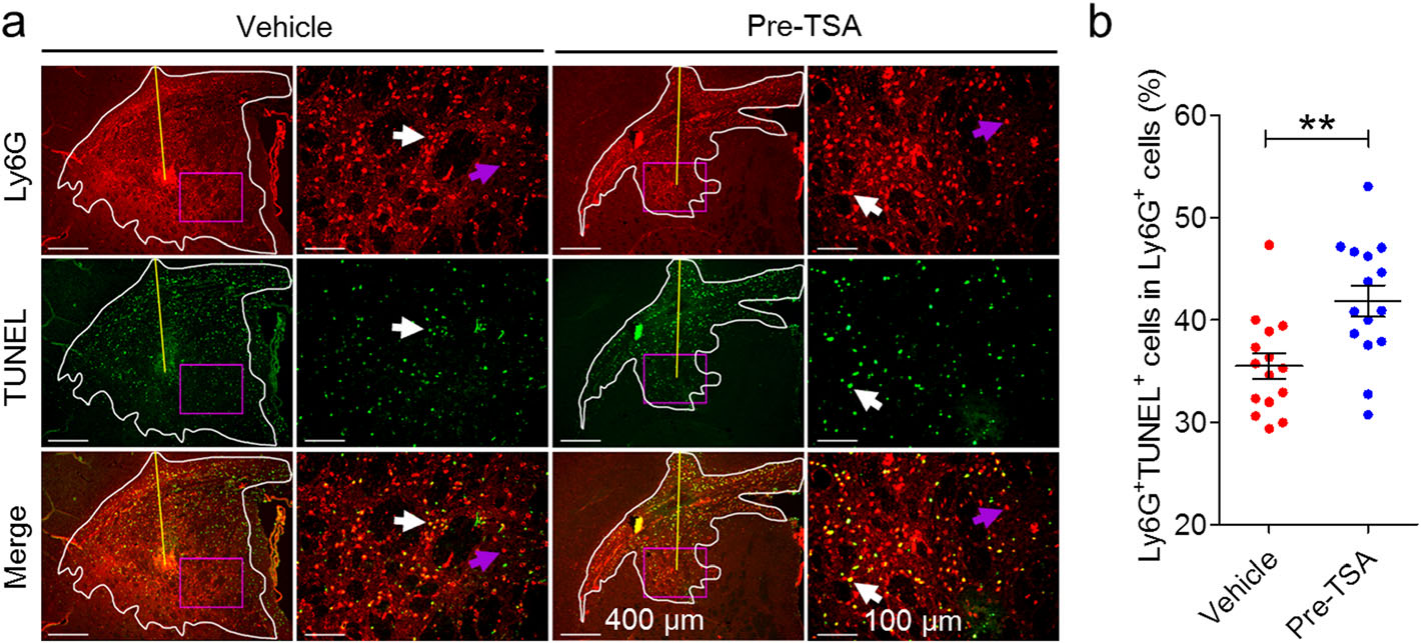
TSA promotes apoptosis of recruited neutrophils in NMOSD lesions at 24 h after brain injection. Ly6G and TUNEL immunofluorescence double staining was performed to determine the extent of spontaneous neutrophil apoptosis in NMOSD lesions at 24 h after brain injection. **a** Representative immunofluorescence staining of Ly6G (*top*, red) and TUNEL (*middle*, green). Yellow lines indicate the needle tract; white lines delimit the lesion; purple rectangles indicate magnified field. White arrows indicate apoptotic neutrophils (Ly6G^+^ TUNEL^+^, yellow); purple arrows indicate nonapoptotic neutrophils (Ly6G^+^ TUNEL^−^, red). **b** Quantification of neutrophil apoptosis was defined as the ratio of Ly6G^+^ TUNEL^+^ cells in Ly6G^+^ cells in the NMOSD lesion-side hemisphere (*n* = 15, **p < 0.01). The data are representative of three independent experiments. Mann-Whitney tests was performed

To clarify whether the protective effect of TSA after injection of NMO-IgG and hC is mediated by promoting the resolution of inflammation, tissues were immunofluorescently stained for TUNEL and the dominant inflammatory cell (neutrophil)-specific marker (Ly6G). We observed that abundant levels of neutrophils were colocalized with TUNEL in NMOSD lesions at 24 h after brain infusion (Fig. 4a). Interestingly, the frequency of apoptotic neutrophils (Ly6G^+^ TUNEL^+^) was substantially higher in mice pretreated with TSA than in mice treated with vehicle (44.33% ± 2.50% versus 32.29% ± 1.75%; Mann-Whitney test, *p* = 0.0079, *n* = 15; Fig. 4b). Thus, these results indicate that TSA-induced neutrophil apoptosis is partially responsible for the beneficial effects of TSA on the mouse NMOSD model.

### TSA induces mouse neutrophil apoptosis via caspase-dependent pathways in vitro

**Fig. 5:**
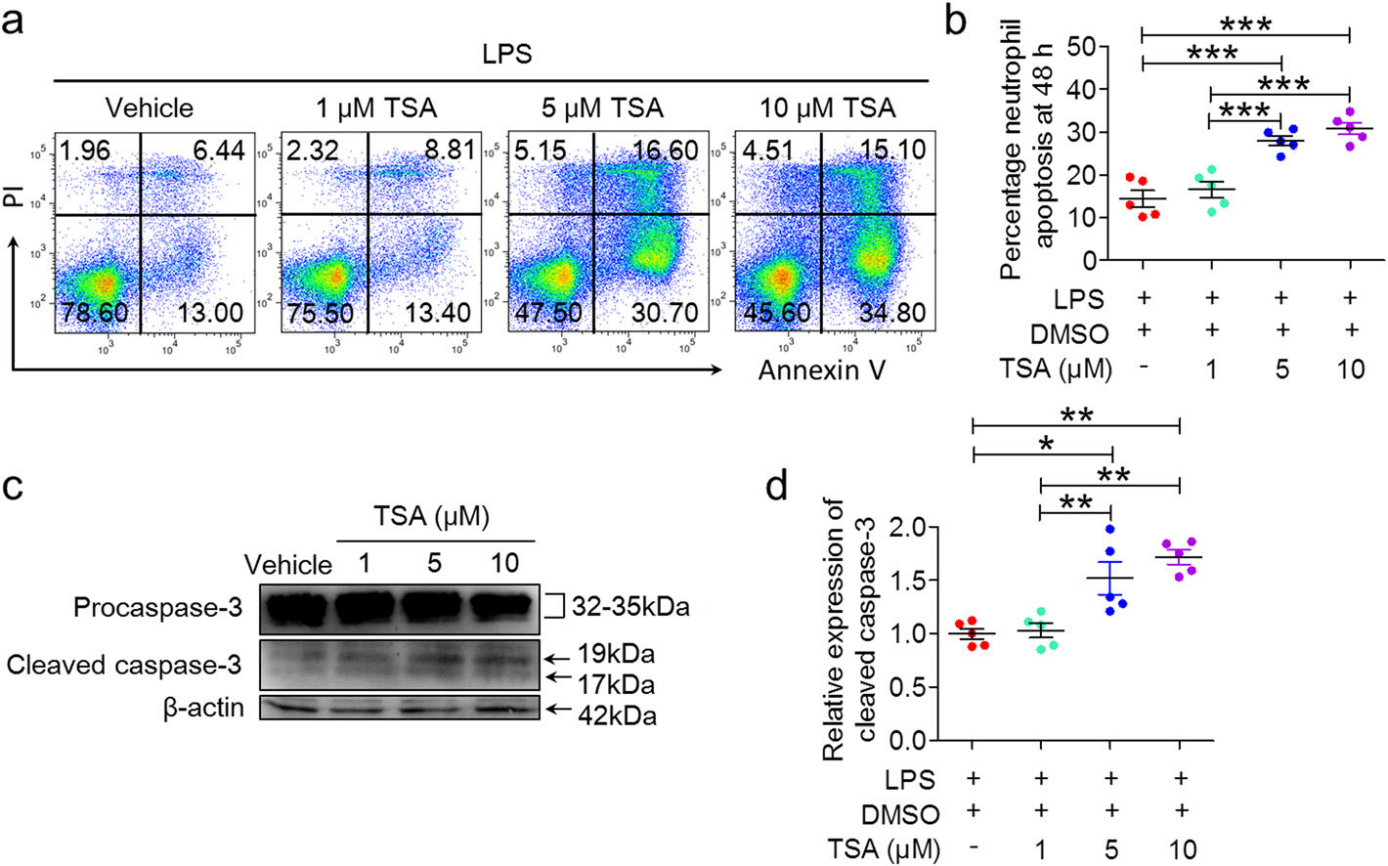
TSA promotes mouse neutrophil apoptosis via caspase-dependent pathways in vitro. Fresh mouse BMNEs (1 × 10^6^ cells/mL/well) were cultured in RPMI 1640 complete medium at 37 °C with 5% CO_2_ and stimulated with 100 ng/mL LPS for 21 h in the absence or presence of TSA (1 μM, 5 μM, and 10 μM). **a**, **b** Neutrophil apoptosis was assessed by flow cytometry after APC-Annexin V/PI labeling. For each condition, 3 × 10^4^ events were recorded within the neutrophil gate. **a** Representative flow cytometry plots showing Annexin-V/PI binding. **b** The apoptosis rate of mouse BMNEs (*n* = 5, ***p < 0.001). **c** Western blotting was performed to analyze procaspase 3 (33 kDa) and cleaved caspase 3 (17 kDa and 19 kDa) expression. **d** Quantification of the relative expression of procaspase 3 and cleaved caspase 3 (*n* = 5, *p < 0.05, **p < 0.01). β-actin was used as an internal control. The data are representative of three independent experiments. One-way ANOVA followed by Tukey’s multiple comparison tests was performed

To investigate whether TSA can directly induce neutrophil apoptosis, freshly isolated mouse BMNEs were incubated with increasing concentrations of TSA in the presence of 100 ng/mL LPS for 48 h, and then viability and apoptosis were analyzed by flow cytometry. Annexin V/PI staining showed that the frequency of apoptotic neutrophils was strikingly suppressed in response to inflammatory stimuli (from 34.2 ± 1.0 to 14.4 ± 2.0%), and the life span of neutrophils was prolonged (Fig. S2a, b). The addition of TSA to neutrophil cultures potently inhibited LPS-induced neutrophil survival and induced neutrophil apoptosis in a concentration-dependent manner. After incubation with TSA (5 μM and 10 μM) in the presence of LPS for 21 h, the frequency of apoptotic neutrophils was significantly elevated (Fig. 5a, b). In addition, western blotting further demonstrated that TSA promoted the cleavage of procaspase-3 in a dose-dependent manner (Fig. 5c, d). Collectively, these data indicate that TSA can promote mouse neutrophil apoptosis under inflammatory conditions via caspase-dependent pathways in vitro.

### TSA also induces human neutrophil apoptosis via caspase-dependent pathways in vitro

**Fig. 6:**
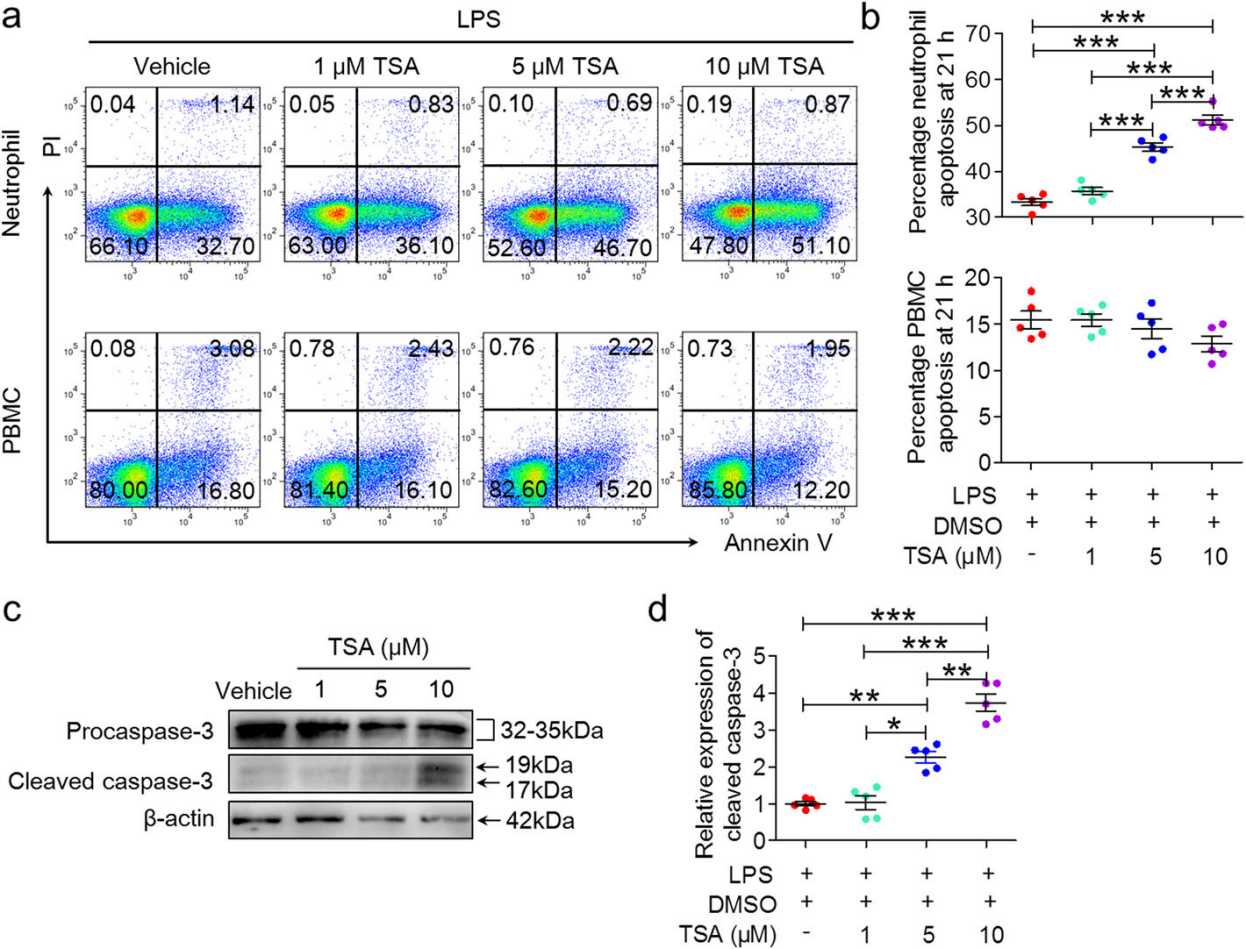
TSA accelerates human neutrophil apoptosis via caspase-dependent pathways in vitro. Human primary neutrophils and PBMCs (1 × 10^6^ cells/mL/well) were plated in 24-well plates at 37 °C with 5% CO_2_ and stimulated with 100 ng/mL LPS for 21 h in the absence or presence of TSA (1 μM, 5 μM, and 10 μM). **a**, **b** Flow cytometry analysis of cultured neutrophils and PBMCs was performed after APC-Annexin V/PI staining. **a** Representative flow cytometry plots showing Annexin-V/PI binding (human neutrophils, *top*; human PBMCs, *bottom*). **b** Quantification of the rate of apoptotic cells (human neutrophils, *top*; human PBMCs, *bottom*; *n* = 5, ***p < 0.001). **c** The cleavage of procaspase-3 was analyzed by western blotting. **d** Quantification of the relative expression of procaspase 3 and cleaved caspase 3 (*n* = 5, *p < 0.05, **p < 0.01, ***p < 0.001). The expression of target proteins was normalized to β-actin. The data are representative of three independent experiments. One-way ANOVA followed by Tukey’s multiple comparison tests was performed

In human neutrophil cultures, we also observed that inflammatory stimuli (100 ng/mL LPS) could dramatically promote neutrophil survival and suppress neutrophil apoptosis (from 74.3 ± 3.1 to 33.3 ± 0.8%) (Fig. S2c, d). After culture with TSA (5 μM and 10 μM) in the presence of LPS for 21 h, the frequency of apoptotic neutrophils was strongly elevated (Fig. 6a, b *top*). Interestingly, TSA had no significant effect on PBMC viability and apoptosis (Fig. 6a, b *bottom*). TSA selectively induced apoptosis in neutrophils but not PBMCs. Similarly, western blotting also showed that TSA significantly enhanced the cleavage of procaspase-3 in a dose-dependent manner (Fig. 6c, d). Taken together, these results suggest that TSA also promotes human neutrophil apoptosis via caspase-dependent pathways in vitro.

### TSA suppresses activation of the NF-κB signaling pathway in vivo

**Fig. 7:**
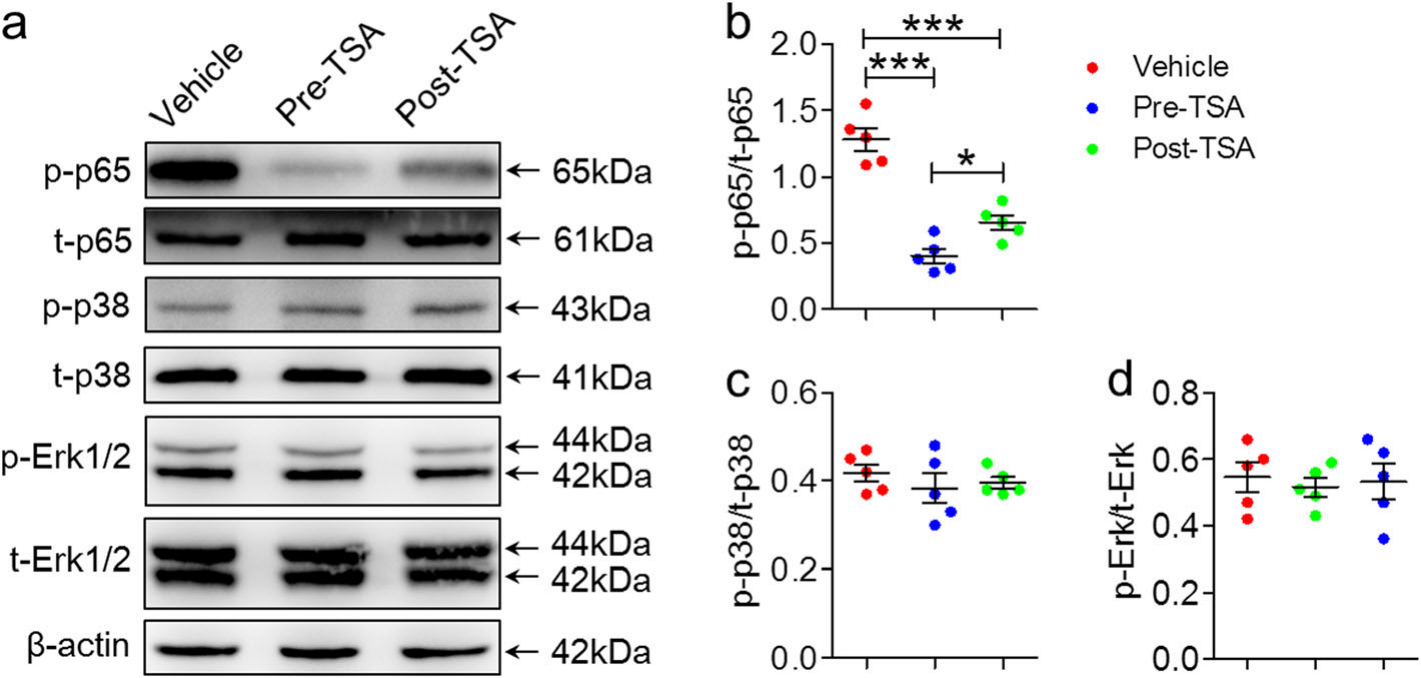
TSA suppressed activation of the NF-κB signaling pathway. Western blotting was performed to analyze the levels of p-p65, t-p65, p-p38, t-p38, p-Erk1/2, and t-Erk1/2 in the hemispheres containing the lesions from TSA- or vehicle-treated NMOSD mice at the end of the experiment. **a** Representative western blots showing p-p65, t-p65, p-p38, t-p38, p-Erk1/2, t-Erk1/2, and β-actin. **b** Quantification of the expression of p-p65 relative to t-p65. **c** Quantification of the expression of p-p38 relative to t-p38. **d** Quantification of the expression of p-Erk relative to t-Erk (*n* = 5). β-actin was used as an internal control. *n* = 5, *p < 0.05, ***p < 0.001. One-way ANOVA followed by Tukey’s multiple comparison tests was performed

To clarify the mechanism of TSA and promote its clinical transformation, it is necessary to evaluate other reported actions of this drug. Previous studies have demonstrated that TSA can inhibit activation of the NF-κB [37–39] and p38 MAPK [37, 38, 40] signaling pathways. Thus, the expression of key molecules of the NF-κB (p-p65 and t-p65) and p38 MAPK (p-p38, t-p38, p-Erk1/2, and t-Erk1/2) signaling pathways were examined in the lesion-side hemispheres from the three groups by western blotting. We found that the relative level of p-p65 to t-p65 was dramatically reduced in the TSA-treated group compared to the control group, and the pre-TSA group displayed a more obvious effect than the post-TSA group (Fig. 7a, b). However, the expression of p-p38, t-p38, p-Erk1/2, and t-Erk1/2 was not changed compared to that of the control group (Fig. 7a, c, d). These results indicate that TSA can inhibit activation of the NF-κB signaling pathway in vivo, which may be related to the therapeutic effect of TSA on NMOSD mice.

## Discussion

Here, we confirmed that neutrophils are the dominant inflammatory cells during the acute phase in relatively early NMOSD model lesions (24 h). TSA can efficiently reduce NMO-IgG-induced damage in the mouse brain, accompanied by an increased frequency of apoptotic neutrophils. These results suggest that the possible mechanisms by which TSA exerts protective effects in this NMOSD model may be by promoting the resolution of inflammation by accelerating neutrophil apoptosis.

To validate the effect of TSA on neutrophils in the NMOSD model, we performed in vitro experiments. We observed that the inflammatory milieu dramatically suppressed neutrophil apoptosis and prolonged the neutrophil lifespan, but TSA partially alleviated this suppression and significantly accelerated apoptosis in primary neutrophils isolated from mouse BM or human peripheral blood. This finding is nearly consistent with a previous finding that TSA can promote the elimination of inflammation by accelerating the process of neutrophil reverse migration and spontaneous apoptosis in a zebrafish sterile injury model [33]. However, the experimental design and methods used in our study make it difficult to discern whether the reduction in neutrophils is partially caused by reverse migration. This is an important question, and future studies are warranted.

The inflammatory cells recruited into NMOSD lesions mainly include neutrophils, macrophages, and eosinophils, with relatively few T lymphocytes [21, 41]. During the pathogenesis of NMOSD, these inflammatory cells represent temporal dynamic changes. Neutrophils are the first cell type to be recruited into NMOSD lesions, which occurs only a few hours after intracerebral injection of NMO-IgG and hC [21]. Within 12 h, there is perivascular complement activation, with the loss of AQP4 and early myelin loss [21, 42]. By 24 h, neutrophils enter the lesion and accumulate around small vessels [21, 42], where AQP4 is highly expressed. At the chronic phase (7 days), few neutrophils remain in the lesions, and macrophages dominate and infiltrate extensively into the white matter [21, 42]. Perivascular eosinophils are also observed at 7 days. However, the role of macrophages and eosinophils in NMOSD lesions is unclear [21]. For these reasons, 24 h postinjection was selected as the detection time point in the present study.

Neutrophils are the most abundant cell type in the innate immune system and have been implicated in the pathogenesis of NMOSD [19, 21, 23]. Under physiological conditions, circulating neutrophils have a very short half-life because they constitutively undergo apoptosis and are functionally quiescent [43, 44]. However, the neutrophil lifespan is dynamically influenced during the course of the inflammatory response and depends on the balance between signals that promote survival and those that accelerate apoptosis [45–47]. After recruitment to injured sites, the lifespan of neutrophils is markedly prolonged by the inflammatory microenvironment, which allows effective phagocytosis and probably causes excessive tissue damage. The ideal result is that neutrophils will undergo spontaneous apoptosis after their physiological function (e.g., elimination of damaged tissue components) has been fulfilled in the injured sites. In contrast to necrosis, apoptosis renders neutrophils unresponsive to extracellular stimuli, prevents the release of toxic constituents, and facilitates their recognition and clearance by macrophages [48, 49]. Thus, neutrophil apoptosis has emerged as a crucial control point in determining the outcome of the inflammatory response. Blocking neutrophil apoptosis produces persistent inflammation, whereas accelerating neutrophil apoptosis enhances the resolution of inflammation to restrain excessive tissue injury and avoid persistent chronic inflammation [44].

As a lipid-soluble compound, TSA can penetrate the BBB and enter the brain parenchyma, partially avoiding the difficulty faced by water-soluble drugs. Sucher et al. reported that TSA was detectable in the brain within 5 min of injection, which is almost the same time it was detected in the circulation. In addition, the concentration of TSA in the brain reached a peak at 60 min, decreased slowly over several hours, and was undetectable after 8 h [50].

Currently, therapeutic strategies for NMOSD mainly focus on limiting the deleterious effects of AQP4-IgG binding [51], complement activation [17, 24, 52, 53], IgG production [54], neutrophil activation [21], and eosinophil activation [55]. Thus, screening small molecule compounds to target these deleterious effects will be a potential approach for NMOSD therapy [19].

## Conclusion

In summary, TSA can act as a prophylactic treatment that reduces NMO-IgG-induced damage in the mouse brain by promoting the resolution of inflammation by inducing neutrophil apoptosis. TSA can also restrict activation of the NF-κB signaling pathway in vivo, which may be involved in the effect of TSA on NMOSD mice. Thus, I believe that further studies are needed to elucidate these mechanisms in the future. Collectively, these findings strongly support the notion that TSA has potent anti-inflammatory functions and may serve as a promising therapeutic agent for human neutrophil-associated inflammatory disorders, such as NMOSD.

## Supplementary information

**Additional file 1: Fig. S1.** Purity test for neutrophils isolated from mouse BM or human peripheral blood. **Fig. S2.** Inflammatory stimuli dramatically suppresses neutrophil spontaneous apoptosis.

## Data Availability

Please contact the author for data requests.

## Abbreviations

NMOSD: Neuromyelitis optica spectrum disorder
AQP4: Aquaporin-4
TSA: Tanshinone IIA
GFAP: Glial fibrillary acidic protein
MBP: Myelin basic protein
CNS: Central nervous system
BBB: Blood-brain barrier
RRMS: Relapsing-remitting multiple sclerosis
IgG: Immunoglobulin G
CDC: Complement-dependent cytotoxicity
ADCC: Antibody-dependent cell-mediated cytotoxicity
hC: Human complement
G-CSF: Granulocyte-colony stimulating factor
Iba-1: Ionized calcium binding adapter molecule 1
BMNE: Bone marrow-derived neutrophil
PBS: Phosphate buffer saline
HBSS: Hank’s balanced salt solution
PBMCs: Peripheral blood mononuclear cells
PMNs: Polymorphonuclear cells
SDS-PAGE: Sodium dodecyl sulfate-polyacrylamide gel
PVDF: Polyvinylidene fluoride
TUNEL: Terminal deoxynucleotidyl transferase (TdT)-mediated biotin-dUTP nick end labeling
PI: Propidium iodide
MAPK: Mitogen-activated protein kinase
Erk: Extracellular-regulated protein kinase

## References

[1] Wingerchuk DM, Lennon VA, Lucchinetti CF, Pittock SJ, Weinshenker BG (2007). The spectrum of neuromyelitis optica. Lancet Neurol.

[2] Jarius S, Paul F, Franciotta D, Waters P, Zipp F, Hohlfeld R (2008). Mechanisms of disease: aquaporin-4 antibodies in neuromyelitis optica. Nat Clin Pract Neurol.

[3] Bennett JL, Lam C, Kalluri SR, Saikali P, Bautista K, Dupree C (2009). Intrathecal pathogenic anti-aquaporin-4 antibodies in early neuromyelitis optica. Ann Neurol.

[4] Kawachi I, Lassmann H (2017). Neurodegeneration in multiple sclerosis and neuromyelitis optica. J Neurol Neurosurg Psychiatry.

[5] Takai Y, Misu T, Takahashi T, Nakashima I, Fujihara K (2013). NMO spectrum disorders and anti AQP4 antibody. Brain Nerve.

[6] Takahashi T, Fujihara K, Nakashima I, Misu T, Miyazawa I, Nakamura M (2007). Anti-aquaporin-4 antibody is involved in the pathogenesis of NMO: a study on antibody titre. Brain.

[7] Lennon VA, Kryzer TJ, Pittock SJ, Verkman AS, Hinson SR (2005). IgG marker of optic-spinal multiple sclerosis binds to the aquaporin-4 water channel. J Exp Med.

[8] Jarius S, Wildemann B (2010). AQP4 antibodies in neuromyelitis optica: diagnostic and pathogenetic relevance. Nat Rev Neurol.

[9] Nielsen S, Nagelhus EA, Amiry-Moghaddam M, Bourque C, Agre P, Ottersen OP (1997). Specialized membrane domains for water transport in glial cells: high-resolution immunogold cytochemistry of aquaporin-4 in rat brain. J Neurosci.

[10] Verkman AS, Phuan PW, Asavapanumas N, Tradtrantip L (2013). Biology of AQP4 and anti-AQP4 antibody: therapeutic implications for NMO. Brain Pathol.

[11] Paul F, Jarius S, Aktas O, Bluthner M, Bauer O, Appelhans H, et al. Antibody to aquaporin 4 in the diagnosis of neuromyelitis optica. PLoS Med. 2007;(4):e133. 10.1371/journal.pmed.0040133 PMC1852124 17439296

[12] Ratelade J, Bennett JL, Verkman AS (2011). Evidence against cellular internalization in vivo of NMO-IgG, aquaporin-4, and excitatory amino acid transporter 2 in neuromyelitis optica. J Biol Chem.

[13] Bradl M, Misu T, Takahashi T, Watanabe M, Mader S, Reindl M (2009). Neuromyelitis optica: pathogenicity of patient immunoglobulin in vivo. Ann Neurol.

[14] Nicchia GP, Mastrototaro M, Rossi A, Pisani F, Tortorella C, Ruggieri M (2009). Aquaporin-4 orthogonal arrays of particles are the target for neuromyelitis optica autoantibodies. Glia..

[15] Takeshita Y, Obermeier B, Cotleur AC, Spampinato SF, Shimizu F, Yamamoto E (2017). Effects of neuromyelitis optica-IgG at the blood-brain barrier in vitro. Neurol Neuroimmunol Neuroinflamm..

[16] Phuan PW, Ratelade J, Rossi A, Tradtrantip L, Verkman AS (2012). Complement-dependent cytotoxicity in neuromyelitis optica requires aquaporin-4 protein assembly in orthogonal arrays. J Biol Chem.

[17] Phuan PW, Zhang H, Asavapanumas N, Leviten M, Rosenthal A, Tradtrantip L (2013). C1q-targeted monoclonal antibody prevents complement-dependent cytotoxicity and neuropathology in in vitro and mouse models of neuromyelitis optica. Acta Neuropathol.

[18] Ratelade J, Asavapanumas N, Ritchie AM, Wemlinger S, Bennett JL, Verkman AS (2013). Involvement of antibody-dependent cell-mediated cytotoxicity in inflammatory demyelination in a mouse model of neuromyelitis optica. Acta Neuropathol.

[19] Zhang H, Verkman AS (2013). Eosinophil pathogenicity mechanisms and therapeutics in neuromyelitis optica. J Clin Invest.

[20] Hertwig L, Pache F, Romero-Suarez S, Sturner KH, Borisow N, Behrens J (2016). Distinct functionality of neutrophils in multiple sclerosis and neuromyelitis optica. Mult Scler.

[21] Saadoun S, Waters P, MacDonald C, Bell BA, Vincent A, Verkman AS (2012). Neutrophil protease inhibition reduces neuromyelitis optica-immunoglobulin G-induced damage in mouse brain. Ann Neurol.

[22] Jacob A, Saadoun S, Kitley J, Leite M, Palace J, Schon F (2012). Detrimental role of granulocyte-colony stimulating factor in neuromyelitis optica: clinical case and histological evidence. Mult Scler.

[23] Saadoun S, Waters P, Bell BA, Vincent A, Verkman AS, Papadopoulos MC (2010). Intra-cerebral injection of neuromyelitis optica immunoglobulin G and human complement produces neuromyelitis optica lesions in mice. Brain..

[24] Shi K, Wang Z, Liu Y, Gong Y, Fu Y, Li S (2016). CFHR1-modified neural stem cells ameliorated brain injury in a mouse model of neuromyelitis optica spectrum disorders. J Immunol.

[25] Wang Z, Guo W, Liu Y, Gong Y, Ding X, Shi K (2017). Low expression of complement inhibitory protein CD59 contributes to humoral autoimmunity against astrocytes. Brain Behav Immun.

[26] Wrzos C, Winkler A, Metz I, Kayser DM, Thal DR, Wegner C (2014). Early loss of oligodendrocytes in human and experimental neuromyelitis optica lesions. Acta Neuropathol.

[27] Fu J, Huang H, Liu J, Pi R, Chen J, Liu P (2007). Tanshinone IIA protects cardiac myocytes against oxidative stress-triggered damage and apoptosis. Eur J Pharmacol.

[28] Yu ML, Li SM, Gao X, Li JG, Xu H, Chen KJ. Sodium tanshinone II a sulfonate for coronary heart disease: a systematic review of randomized controlled trials. Chin J Integr Med. 2018. 10.1007/s11655-018-2556-7 29752695

[29] Yan FF, Liu YF, Liu Y, Zhao YX (2009). Sulfotanshinone sodium injection could decrease fibrinogen level and improve clinical outcomes in patients with unstable angina pectoris. Int J Cardiol.

[30] Ji B, Zhou F, Han L, Yang J, Fan H, Li S (2017). Sodium tanshinone IIA sulfonate enhances effectiveness Rt-PA treatment in acute ischemic stroke patients associated with ameliorating blood-brain barrier damage. Transl Stroke Res.

[31] Chen L, Bi XY, Zhu LX, Qiu YQ, Ding SJ, Deng BQ (2011). Flavonoids of puerarin versus tanshinone II a for ischemic stroke: a randomized controlled trial. Zhong Xi Yi Jie He Xue Bao.

[32] Xu G, Zhao W, Zhou Z, Zhang R, Zhu W, Liu X (2009). Danshen extracts decrease blood C reactive protein and prevent ischemic stroke recurrence: a controlled pilot study. Phytother Res.

[33] Robertson AL, Holmes GR, Bojarczuk AN, Burgon J, Loynes CA, Chimen M (2014). A zebrafish compound screen reveals modulation of neutrophil reverse migration as an anti-inflammatory mechanism. Sci Transl Med.

[34] Zhang S, Huang G, Yuan K, Zhu Q, Sheng H, Yu R (2017). Tanshinone IIA ameliorates chronic arthritis in mice by modulating neutrophil activities. Clin Exp Immunol.

[35] Sun Y, Abbondante S, Karmakar M, de Jesus CS, Che C, Hise AG (2018). Neutrophil caspase-11 is required for cleavage of caspase-1 and secretion of IL-1beta in aspergillus fumigatus infection. J Immunol.

[36] McNaughton EF, Eustace AD, King S, Sessions RB, Kay A, Farris M (2018). Novel anti-inflammatory peptides based on chemokine-glycosaminoglycan interactions reduce leukocyte migration and disease severity in a model of rheumatoid arthritis. J Immunol.

[37] Wang Z, Li J, Zhang J, Xie X (2019). Sodium tanshinone IIA sulfonate inhibits proliferation, migration, invasion and inflammation in rheumatoid arthritis fibroblast-like synoviocytes. Int Immunopharmacol.

[38] Yin X, Yin Y, Cao FL, Chen YF, Peng Y, Hou WG (2012). Tanshinone IIA attenuates the inflammatory response and apoptosis after traumatic injury of the spinal cord in adult rats. PLoS One.

[39] Quan M, Lv Y, Dai Y, Qi B, Fu L, Chen X (2019). Tanshinone IIA protects against lipopolysaccharide-induced lung injury through targeting Sirt1. J Pharm Pharmacol.

[40] Liu X, Ye M, An C, Pan L, Ji L (2013). The effect of cationic albumin-conjugated PEGylated tanshinone IIA nanoparticles on neuronal signal pathways and neuroprotection in cerebral ischemia. Biomaterials..

[41] Roemer SF, Parisi JE, Lennon VA, Benarroch EE, Lassmann H, Bruck W (2007). Pattern-specific loss of aquaporin-4 immunoreactivity distinguishes neuromyelitis optica from multiple sclerosis. Brain..

[42] Saadoun S, Waters P, Macdonald C, Bridges LR, Bell BA, Vincent A (2011). T cell deficiency does not reduce lesions in mice produced by intracerebral injection of NMO-IgG and complement. J Neuroimmunol.

[43] Savill J, Dransfield I, Gregory C, Haslett C (2002). A blast from the past: clearance of apoptotic cells regulates immune responses. Nat Rev Immunol.

[44] Gilroy DW, Lawrence T, Perretti M, Rossi AG (2004). Inflammatory resolution: new opportunities for drug discovery. Nat Rev Drug Discov.

[45] Luo HR, Loison F (2008). Constitutive neutrophil apoptosis: mechanisms and regulation. Am J Hematol.

[46] Simon HU (2003). Neutrophil apoptosis pathways and their modifications in inflammation. Immunol Rev.

[47] El Kebir D, Filep JG (2010). Role of neutrophil apoptosis in the resolution of inflammation. ScientificWorldJournal..

[48] Savill JS, Wyllie AH, Henson JE, Walport MJ, Henson PM, Haslett C (1989). Macrophage phagocytosis of aging neutrophils in inflammation. Programmed cell death in the neutrophil leads to its recognition by macrophages. J Clin Invest.

[49] Renshaw SA, Parmar JS, Singleton V, Rowe SJ, Dockrell DH, Dower SK (2003). Acceleration of human neutrophil apoptosis by TRAIL. J Immunol.

[50] Lam BY, Lo AC, Sun X, Luo HW, Chung SK, Sucher NJ (2003). Neuroprotective effects of tanshinones in transient focal cerebral ischemia in mice. Phytomedicine..

[51] Tradtrantip L, Zhang H, Saadoun S, Phuan PW, Lam C, Papadopoulos MC (2012). Anti-aquaporin-4 monoclonal antibody blocker therapy for neuromyelitis optica. Ann Neurol.

[52] Zhu W, Wang Z, Hu S, Gong Y, Liu Y, Song H (2019). Human C5-specific single-chain variable fragment ameliorates brain injury in a model of NMOSD. Neurol Neuroimmunol Neuroinflamm..

[53] Paul F, Murphy O, Pardo S, Levy M (2018). Investigational drugs in development to prevent neuromyelitis optica relapses. Expert Opin Investig Drugs.

[54] Hodecker SC, Stellmann JP, Rosenkranz SC, Young K, Holst B, Friese MA (2017). Ruxolitinib treatment in a patient with neuromyelitis optica: a case report. Neurol Neuroimmunol Neuroinflamm..

[55] Katz Sand I, Fabian MT, Telford R, Kraus TA, Chehade M, Masilamani M (2018). Open-label, add-on trial of cetirizine for neuromyelitis optica. Neurol Neuroimmunol Neuroinflamm.

