# Supplementary material for: Tanshinone IIA alleviates brain damage in a mouse model of neuromyelitis optica spectrum disorder by inducing neutrophil apoptosis
Source: J Neuroinflammation. 2020 Jun 25;17:198. doi: 10.1186/s12974-020-01874-6 (PMC7318433; doi:10.1186/s12974-020-01874-6)
Supplement: Supplementary file 1 — Additional file 1: Fig. S1. Purity test for neutrophils isolated from mouse BM or human peripheral blood. Fig. S2. Inflammatory stimuli dramatically suppresses neutrophil spontaneous apoptosis. [file 12974_2020_1874_MOESM1_ESM.docx]

**Supplementary material**

**
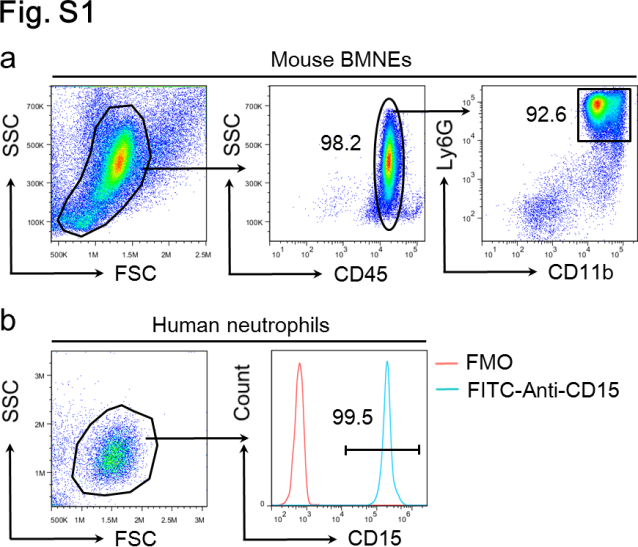
**

**Fig. S1** Purity test for neutrophils isolated from mouse BM or human peripheral blood. **a** Neutrophils were prepared as described in Materials and Methods. Then, the purity of mouse BMNEs was confirmed by flow cytometry analysis using APC-anti-CD45, Alexa Flour 488-anti-CD11b and PE-anti-Ly6G antibody staining. The purity of mature mouse neutrophils was identified as CD11b^hi^ Ly6G^hi^ cells in the CD45^+^ gate (purity = 90.93%). **b** Human neutrophils were isolated from peripheral blood as described above. The purity of neutrophils was determined by flow cytometry analysis using FITC-CD15 staining and identified as CD15^hi^ subsets in all cells (purity = 99.5%). The solid red line depicts fluorescence-minus-one (FMO) control, the solid cyan line represents FITC-labeled anti-human-CD15 antibody staining, and the numbers indicate the purity of mouse BMNEs.


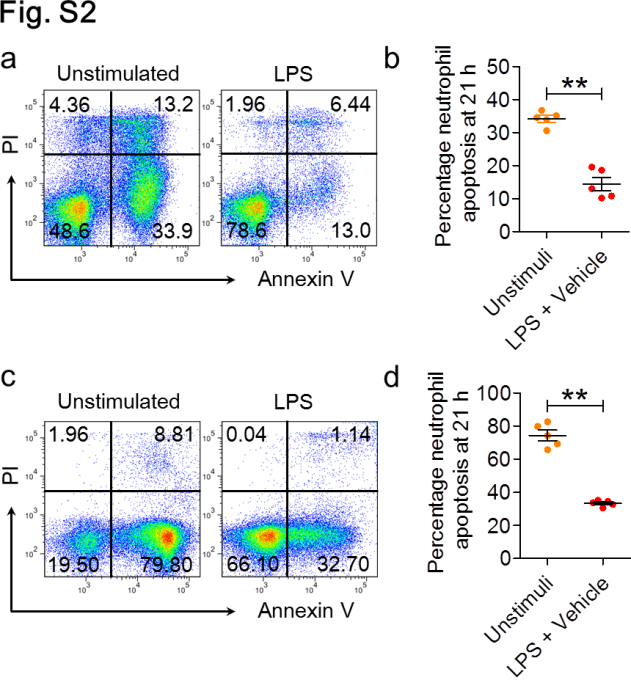


**Fig. S2** Inflammatory stimuli dramatically suppresses neutrophil spontaneous apoptosis. Fresh neutrophils (1×10^6^ cells/mL/well) were cultured in RPMI 1640 complete medium at 37°C with 5% CO_2_ in the presence or absence of 100 ng/mL LPS for 21 h. Neutrophil apoptosis was assessed by flow cytometry after allophycocyanin-Annexin V/PI labeling. **a** Representative flow cytometry plots of Annexin-V/PI binding for mouse neutrophils. **b** Quantification of the rate of apoptotic mouse neutrophils (mean ± SEM, n = 5, *p* = 0.0079, Mann-Whitney test). **c** Representative flow cytometry plots of Annexin-V/PI binding for human neutrophils. **d** The apoptosis rate of human neutrophils (mean ± SEM, n = 5, *p* = 0.0079, Mann-Whitney test). Data are representative of three independent experiments, *p* < 0.05 was considered statistically significant.
